# Bibliographical Notices

**Published:** 1847-02

**Authors:** 


### BIBLIOGRAPHICAL NOTICES.

*Encyclopedia Americana; Supplementary Volume, a popular Dictionary of Arts, Sciences, Literature, History, Politics, and Biography*, vol. xiv., Edited by HENRY VETHAKE, L. L. D., Vice-Provost and Prof. Mathematics in the University of Pennsylvania, etc., Philadelphia, Lea & Blanchard, 1847, 8 vo. pp. 662.

The design of this volume is set forth by the Editor in the following quotation from the preface :

"The Encyclopædia Americana, to which the present volume is supplementary, has been nearly fourteen years before the public, and has obtained a high rank, in its estimation, among works of a similar nature. It was founded on the basis of the seventh edition of the famous "Conversations-Lexikon." The eighth edition of the latter appeared between the years 1833 and 1837, and the ninth is now in course of publication. Hence it became desirable that this Encyclopædia should be extended, to embrace the improvements thus introduced into the German work. But independently of any information which might be derived from this quarter, how many important events have occurred, or facts been observed, and how many individuals have emerged from comparative obscurity during the lapse of fourteen years,—affording abundant materials for such a volume as is now offered to the public, and even imperatively requiring its publication to restore to the Encyclopædia Americana all the advantages which belonged to it originally, as a book for ready consultation on subjects of general or popular interest!"

The estimation in which the Encyclopædia Americana is held, is sufficiently attested by the fact that the ninth edition of so extensive a work is now called for. The progressive advancement of the arts and sciences, and the constant development of events of history, biography, &c., incident to the lapse of time, render supplemental additions to a work of that character, after several years have elapsed, essential to its continued usefulness. So far as we are authorised to judge from a brief, general examination of the new volume by Prof. Vethake, it supplies very satisfactorily what is needed for the original work to be posted up to the present period. In size and typographical appearance it corresponds with the previous volumes. They who possess, or may purchase the original work, will, of course, desire to have this supplementary dictionary.

---

*Materia Medica and Therapeutics; Including the Preparations of Pharmacopœias of London, Edinburg, Dublin, and [of the United States] with many new medicines. By* J. Forbes Royle, M. D., F, R. S., *late of the Medical Staff of the Bengal Army, Prof. Mat. Med. and Therapeutics, King's College, London, &c. &c. Edited by* Joseph Carson, M. D., *Prof. of Mat. Med. in the Philadelphia College of Pharmacy, &c. &c. With ninety-eight illustrations.* Philadelphia: Lea & Blanchard. 1847. 8vo., pp. 689.

A new text book of Materia Medica may seem hardly called for, so soon after the publication of the admirable work of Dr. Pereira. The author of the volume the title-page of which we have given, states that he was induced to prepare it at the repeated request of an intelligent publisher, who is well acquainted with the wants of the profession, as well as from a conviction, arising from his own experience as a teacher, that the necessities of pupils were not fully provided for by the text-books in common use.

We are not prepared to institute a critical examination between the merits of the two works; but it strikes us that Dr. Royle has succeeded in fulfilling his plan, which, as expressed in his own language, was, to prepare a work "sufficiently full for information, and yet as short and condensed as was compatible with the avoidance of being superficial." While it is less extensive than that of Dr. Pereira, it appears to be sufficiently comprehensive for purposes of practical reference; and this being the case, its conciseness is, in many respects, an advantage.

The arrangement of subjects, and the relative spaces allotted to his leading divisions, are as follows: after devoting a few pages to some judicious introductory considerations, he considers the operations of Pharmacy and Pharmaceutic Chemistry in the next sixteen pages. He then takes up—first, the Mineral Materia Medica, occupying one hundred and eighty-eight pages; second, Vegetable Materia Medica, three hundred and eighty-nine pages. The remainder of the treatise embraces, *Products of Fermentation, Etherification, Acetous Fermentation and Destructive Distillation, Animal Materia Medica;* and, in conclusion, seventeen pages are devoted to the "*Physiological and Therapeutical Arrangement of the Materia Medica.*"

In the portion of the work occupied with Materia Medica proper, the history, physical and chemical properties, preparation, tests, actions, uses and doses, of all the mineral remedies in use, are briefly given; and of vegetable medicines, the botanic classification, physiology, geography, medical properties, together with directions for the collection and drying of vegetables.

"The labors of the editor" (quoting from his preface) "have been confined to the supervision of the work in passing through the press, and the addition of such matter in connection with the Pharmacopœia, and indigenous Materia Medica of the United States, as would render the work fitted for American students and practitioners."

In typographical appearance, the work is on a par with the other medical publications emanating from the enterprising house of Lea & Blanchard.

*Lecture introductory to the course on the Theory and Practice of Medicine in the Medical Department of Pennsylvania College, Session of* 1846—7, *by* W. DARRACK, M. D.

The subject of this discourse is *Life*. Prof. D. treats of this sublime theme, not alone in its physiological and pathological aspects, but in its relations to Psycology and Theology. The reader will find in it profound philosophy, and will rise from its perusal deeply impressed with the learning, eloquence, and devotional spirit of the author.

---

*Lecture, introductory to a course, on Obstetrics and the Diseases of Women aud Children.* By GUNNING S. BEBFORD, M. D., Prof. of Obstet. &c. in the University of New York. Delivered Oct. 30, 1346.

Prof. Bedford is an agreeable writer, and, if we may judge from this and previous introductory lectures which have been published, possesses the tact of communicating information in a clear and forcible manner. The present discourse abounds in useful suggestions, some of which we should transfer to our columns, were it not that we presume it will be extensively disseminated and perused entire.

---

*Fevers, their Diagnosis, Pathology, and Treatment, from the essays on fever in Tweedies' Library of Practical Medicine.* Edited by MEREDITH CLYMER M. D., Prof. of principles and Practice of Medicine in the Franklin Med. Coll. of Philadelphia.

We have, in a previous number, referred to this work, and expressed our intention to make it the subject of an extended analytical review. As the Essays taken from Tweedies' Library have been for some time before the profession, we designed to have confined our analysis chiefly to the additions and annotations by the American Editor. Other duties, however, have prevented us from carrying our intention into effect.

In the meanwhile the book is losing its title to newness, and at the same time,its claims to be enrolled among the classical works on Fever are doubtless becoming properly appreciated. We embrace this occasion to say, that while we should have been better satisfied had Prof. Clymer put forth an entirely original treatise, we can conscientiously recommend the work as a candid and comprehensive digest of our present knowledge of febrile diseases. One-half of the volume is contributed by Prof. Clymer. The original essays are by Drs. Christison, Shapter, Burrows, Gregory, and Locock.

---

*Books shortly to be published.*—Jones on the Eye, and Vogel's Pathological Anatomy, with plates &c., will shortly be issued from the press of Lea & Blanchard, Philadelphia.

In addition to the new works which have been noticed in this No. we have received Murphy on Parturition, and Bennet on the Uterus. An analytical review of the latter will appear in our next No. The former will also be reviewed or noticed in the same number.

---

*La Lancette Canadienne;* Journal Medico-Chirurgical, Public à Montreal, par le Docteur J. L. Zephrohon.

This is the title of a new Journal, in newspaper form, which is issued bi-weekly, at Montreal, in the French language. We are happy to include it among our Exchanges, and would recommend it to those who wish an exercise for keeping up their knowledge of French. The subscription price is Four Dollars per annum. The address of the Proprietors is M. M. Lovell et Gibson, Rue St. Nicholas, Montreal.

---

*Introductory Lecture, delivered before the class of the Baltimore College of Dental Surgery, at the session of* 1846-'7; furnished for publication at the request of the class. By A. Westcott, M. D., D. D. S., Prof. of Operative and Mechanical Dentistry.

Prof. W. discusses, in this lecture, the scientific character of the dental department of medicine and surgery, and the importance of institutions devoted especially to that department. The author is evidently a scholar, as well as (we doubt not) an excellent practitioner and teacher of Dentistry.

